# Biosynthesis of Silver Nanoparticles Using *Stenocereus queretaroensis* Fruit Peel Extract: Study of Antimicrobial Activity

**DOI:** 10.3390/ma14164543

**Published:** 2021-08-12

**Authors:** Eduardo Padilla-Camberos, Ivan Moises Sanchez-Hernandez, Omar Ricardo Torres-Gonzalez, Patricia Ramirez-Rodriguez, Emmanuel Diaz, Holger Wille, Jose Miguel Flores-Fernandez

**Affiliations:** 1 Unit of Medical and Pharmaceutical Biotechnology, Center for Research and Assistance in Technology and Design of the State of Jalisco, A.C. (CIATEJ), Guadalajara 44270, Jalisco, Mexico; isanchez@ciatej.mx (I.M.S.-H.); polimerasados@gmail.com (O.R.T.-G.); acineto123@hotmail.com (P.R.-R.); ediaz@ciatej.mx (E.D.); 2 Department of Biochemistry & Centre for Prions and Protein Folding Diseases, University of Alberta, 204 Brain and Aging Research Building, Edmonton, AB T6G 2M8, Canada; wille@ualberta.ca; 3 Department of Research and Innovation, Universidad Tecnológica de Oriental, Oriental 75020, Puebla, Mexico

**Keywords:** nanoparticles, biosynthesis, antimicrobial activity, *Stenocereus queretaroensis* peel extract, pitaya peel

## Abstract

The synthesis and application of nanomaterials as antioxidants and cytotoxic agents has increased in recent years. Biological methods go beyond the chemical and physical synthesis that is expensive and not friendly to the environment. Foodborne pathogens and microorganisms causing candidiasis are responsible of 5–10% hospitalized patients. The nutritional properties of the fruit called pitaya, from the *Stenocereus queretaroensis* species, have been little explored. Therefore, in this study the phytochemical composition of *S. queretaroensis* peel was evaluated and silver nanoparticles (AgNPs) were synthesized biologically in an environmentally friendly way by *S. queretaroensis* peel aqueous extract that contains phytochemicals capable of reducing silver nitrate. The antimicrobial activity of the AgNPs was tested by determining the minimum inhibitory concentration (MIC), minimal bactericidal concentration (MBC) and time-kill kinetics. AgNPs were characterized visually, by UV-visible spectroscopy and TEM. FTIR spectroscopy identified metabolites responsible for the AgNPs formation. AgNPs showed potent antimicrobial activity against gram-negative and gram-positive bacteria, against fungi, and a methicillin-resistant strain of *S. aureus*. MIC and MBC values were as low as 0.078 and 0.156 μg/mL using AgNPs biosynthesized by *S. queretaroensis* fruit peel and the time kill assay started a log reduction in CFU/mL at 1 × MIC and 2 × MIC. *S. queretaroensis*-mediated AgNPs could be the basis for the formulation of biofilms for packaging products or as disinfectants for use on different surfaces.

## 1. Introduction

Over the years, interest in the development of nanomaterials has increased exponentially, in particular the use of silver nanoparticles (AgNPs), because of its applications as antimicrobials, antioxidants and cytotoxics [1,2]. Silver nanoparticles are synthetized through chemical and physical methods. However, those methods require expensive equipment and are not environmentally friendly due to the toxic chemicals that are used in their production. The biological method stands out as a variant for the generation of nanoparticles (NP) with plant extracts, since the waste generated presents a lesser impact on the environment, energy, and resources, being, in general, an easy, fast and economical process [3].

The antimicrobial activity of AgNPs biosynthesized with different plant extracts has become of great interest to the industry, as bacteria that can be found in food, and inert surfaces that we touch can cause poisonings that are harmful to human health. About 5–10% of hospitalized patients in intensive care suffer from infections due to food poisoning. Some Enterobacteriaceae such as *Enterobacter cloacae*, *Escheríchía coli* and *Salmonella spp.*, as well as gram-positive bacteria, such as *Clostrídíum perfringens* and *Clostridium botulinum*, and gram-positive cocci (*Staphylococcus aureus*), which can be found almost anywhere, are responsible for such infections [4].

A search for antimicrobials that can be used against these bacteria on both living and inert surfaces, and which also do not generate resistance in a short time, is of paramount importance as biosynthesized nanoparticles have variable effects depending on the compounds of the source (plant, fungi, weed, lichen, etc.) used as reducers [5].

Fruit peels have been used in a few studies to biosynthesize silver nanoparticles, but the distinctive band of silver nanoparticle biosynthesis in the UV-visible spectrum around 410–450 nm is missing from existing reports [6]. Furthermore, cladodes from *Opuntia* spp. and the *Ferocactus echidne* plants belonging to the Cactaceae (cactus) family have been used for biosynthesis of silver nanoparticles [7,8]. However, as of yet, there is no report of cactus fruit peel for this purpose. *Stenocereus queretaroensis* is another cactus species with a wide distribution in the Americas, particularly in Mexico, where its fruit, known as pitaya, has been part of the diet of the inhabitants of semi-arid regions since pre-Hispanic times [9]. Commercial use of the pitaya has not been studied due to a general lack of knowledge regarding its nutritional properties. Its composition is characterized by the presence of water-soluble nitrogenous pigments named as betalains that are divided into two structural groups, betacyanins, which give it its color from reddish to violet and betaxanthins conferring a yellow-orange coloration [10]. These pigments possess antioxidant, cytotoxic and anti-inflammatory properties. Moreover, the presence of ascorbic acid (vitamin C) and reducing sugars in the peel [11] is of special interest in accordance with other authors due to their role as silver reducers in the green synthesis of nanoparticles [12]. Nevertheless, the information on the composition of the pitaya peel is very limited; therefore, in this study its phytochemical composition has been evaluated and, for the first time, the synthesis and characterization of silver nanoparticles using an aqueous extract of *S. queretaroensis* fruit peel is reported. In addition, the antimicrobial activity of the synthesized AgNPs against strains of industrial and medical interest was evaluated.

## 2. Materials and Methods

### 2.1. S. queretaroensis Peel Extract

**Scheme 1:**
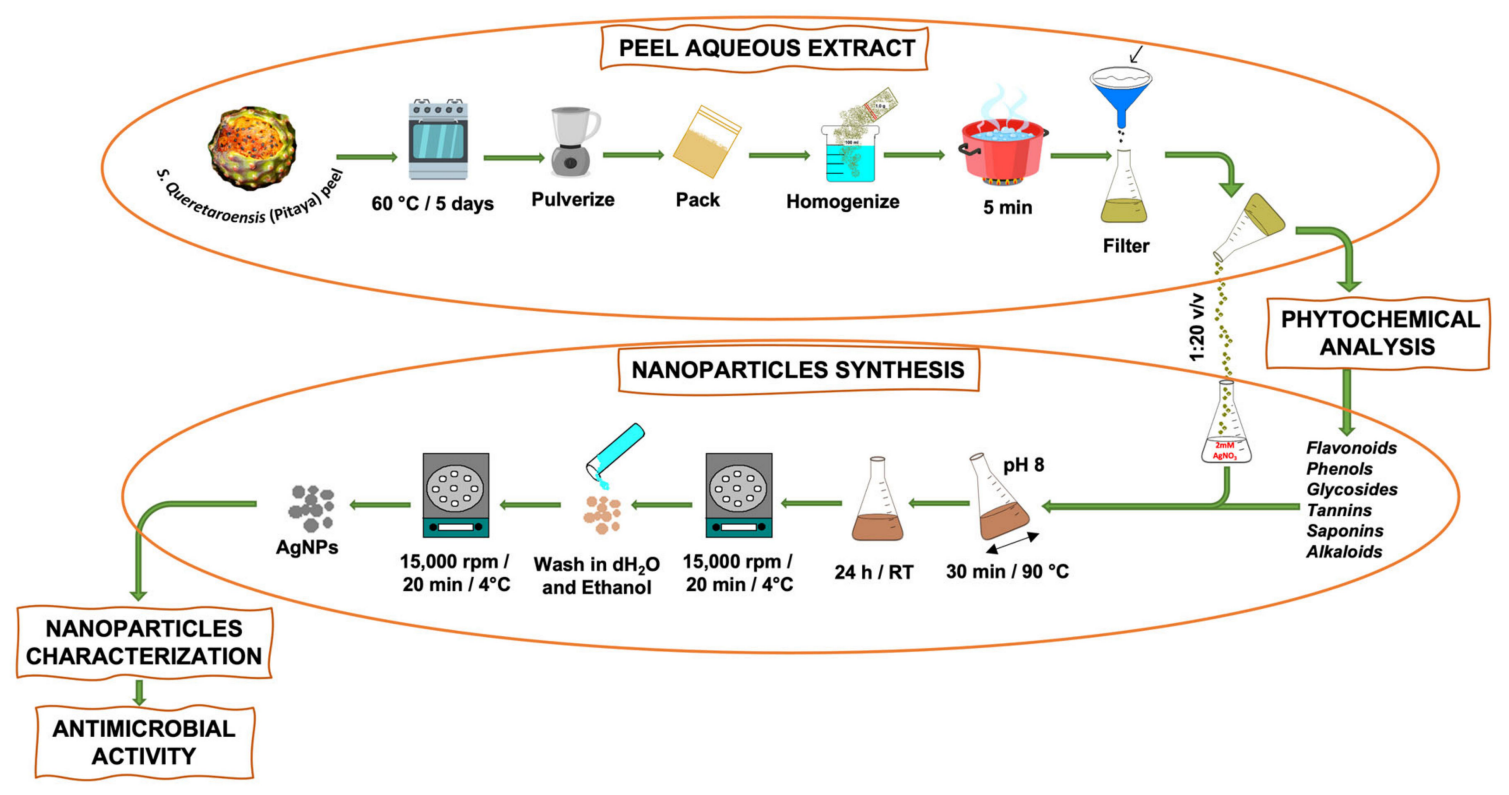
The process of obtaining the *S. queretaroensis* peel aqueous extract and the biosynthesis of *S. queretaroensis*-mediated silver nanoparticles are depicted in a schematic diagram.

*S. queretaroensis* fruit was purchased from a market in Jalisco, Mexico. The extract was obtained according to the methodology published by Loo et al. [13] with some modifications. Briefly, the pitaya peels were dried at 60 °C for 5 days in a stove and then crushed and pulverized by a blender (Hamilton Beach, Henrico County, VA, USA), and finally packed and stored at room temperature (RT); 1.0 g of *S. queretaroensis* dehydrated peel was homogenized in 100 mL of distilled water and was kept boiling for 5 min, and then filtered on Whatman 40 at RT to remove particles and prevent organic matter (Scheme 1). Finally, the *S. queretaroensis* peel aqueous extract was placed in an amber container and kept at 4 °C until further use.

### 2.2. Phytochemical Analysis of S. queretaroensis Peel Aqueous Extract

The presence of bioactive compounds of the *S. queretaroensis* peel aqueous extract was determined by qualitative analysis of flavonoids, phenols, glycosides, tannins, saponins and alkaloids. Flavonoids were determined by aluminum chloride and phenols by Folin-Ciocalteu’s method as described by Marinova et al. [14]. Moreover, glycosides and tannins were determined by Fehling’s method [15]. Saponins were determined according to the method described by Ejikeme et al. [16] and alkaloids by Dragendorff’s method according to Rivas et al. [9] with modifications. Tests were carried out in triplicate.

### 2.3. Synthesis of Silver Nanoparticles

Silver nanoparticles were synthesized by adding *S. queretaroensis* peel aqueous extract to a 2 mM silver nitrate (AgNO_3_) solution at a volume ratio of 1:20. The pH was adjusted to 8 using 1M NaOH. The mixture was stirred for 30 min at a temperature of 90 °C and subsequently allowed to cool and stand for 24 h at room temperature to end the reaction. The formation of colloidal AgNPs was observed by the color change to reddish brown. Subsequently, the nanosilver solution was centrifuged at 15,000× *g* rpm for 20 min at 4 °C and supernatant was discarded. Finally, the pellet was washed with distilled water five times to remove impurities and once with 90% ethanol to obtain pure *S. queretaroensis*-mediated AgNPs (Scheme 1).

### 2.4. Characterization of S. queretaroensis-Mediated Silver Nanoparticles

#### 2.4.1. UV-Visible Spectroscopy

Bioreduction of the AgNO_3_ to *S. queretaroensis*-mediated AgNPs was analyzed using UV-Vis Spectrophotometer (xMark, Bio-Rad, Hercules, CA, USA). Samples were loaded into a 1 cm path length quartz cuvette. The resolution of the equipment was one nm, and a scan in the wavelength range of 300 to 700 nm was carried out.

#### 2.4.2. Fourier Transform Infrared Spectroscopy (FTIR)

Biomolecules responsible for the biosynthesis of *S. queretaroensis*-mediated AgNPs were identified by infrared spectroscopy; 30 μL of sample was placed on an Agilent Cary 630 FTIR Spectrometer (Agilent Technologies, Santa Clara, CA, USA). Transmittance measurements of the *S. queretaroensis* peel aqueous extract and pure AgNPs were carried out in the 4000–400 cm^−1^ range, at a resolution of 4 cm^−1^. MicroLab software (Agilent Technologies, Santa Clara, CA, USA) was used to ascertain peak values.

#### 2.4.3. Dynamic Light Scattering (DLS)

The particle size distribution of *S. queretaroensis*-mediated AgNPs was measured by DLS. Measurement was repeated in triplicate at 25 °C and performed using a Zetasizer equipment (Nano ZS, Malvern, UK).

#### 2.4.4. Transmission Electron Microscopy (TEM)

5-μL drops of nanoparticles in colloidal solution, diluted in distillated water (1:50) and in its pure form were dropped onto freshly glow-discharged 400 mesh carbon coated copper grids (Electron Microscopy Sciences, Hatfield, PA, USA) and were allowed to dry at room temperature.

The morphology and the size distribution of the biosynthesized silver nanoparticles were evaluated by transmission electron microscopy using a Tecnai G20 instrument (FEI Company, Hillsboro, OR, USA) operated at an acceleration voltage of 200 kV. Electron micrographs were acquired with an Eagle 4k × 4k CCD camera (FEI Company, Hillsboro, OR, USA) at nominal magnifications of 11,500×, 50,000× and 62,000×.

### 2.5. Antimicrobial Activity

The minimum inhibitory concentration (MIC), minimal bactericidal concentration (MBC) and Time-kill methods [17] were performed to evaluate the antimicrobial activity of *S. queretaroensis*-mediated AgNPs.

#### 2.5.1. Minimum Inhibitory Concentration (MIC) Determination

Gram-negative bacteria (*Escherichia coli* ATCC 11229, *Salmonella enterica* ATCC 10708, *Pseudomonas aeruginosa* ATCC 9027), gram-positive bacteria (*Staphylococcus aureus* ATCC 6538, methicillin-resistant *S. aureus* (MRSA) ATCC 43300), and the mold *Candida albicans* ATCC 10231 were purchased from American Type Culture Collection (Rockville, MD, USA), and cultured in Mueller Hinton broth (Merk, Germany) at 37 °C and 200 rpm for 24 h. 1 mL bacterial inoculum was inoculated at 1.5 × 10^8^ CFU/mL in 24-well polystyrene plates. Then, eight serial 2-fold dilutions were prepared from the concentration of 10 µg/mL *S. queretaroensis*-mediated AgNPs and of the *S. queretaroensis* peel aqueous extract and 0.5% DMSO (dimethylsulfoxide) was used as negative growth control and MHB broth plus bacterial inoculum as growth control. Antibiotics according to the CLSI guidelines [18] as positive controls were used to validate the test. The plate was incubated at 37 °C for 24 h and the MIC value was determined as the last serial dilution where no visible bacterial growth was noticed by turbidity and color change. Tests were carried out in triplicate.

#### 2.5.2. Minimal Bactericidal Concentration (MBC) Determination

The MBC was determined from the last three wells that showed no bacterial growth from MIC test. Then, 10 μL from the corresponding wells were plated on nutritive agar plates. The plates were incubated at 37 °C for 24 h. The MBC was determined as the lowest concentration of the *S. queretaroensis*-mediated AgNPs or *S. queretaroensis* peel aqueous extract that prevented growth of more than one CFU. Tests were carried out in triplicate.

#### 2.5.3. Time-Kill Kinetics Assay

100 μL of inoculum of each strain at 0.5 of the McFarland scale (1.5 × 10^8^ CFU/mL) was mixed with biosynthesized AgNPs diluted in nutrient broth at concentrations of 0 × MIC, ½ × MIC, 1 × MIC and 2 × MIC (MIC values were obtained from the experiments described above in Section 2.5.1) at final volume of 1 mL, and then incubated at 37 °C at 150 rpm. Subsequently, 100 μL of each culture was spread on nutrient agar plates at different time points (0, 15, 30, 60 and 120 min) and incubated overnight at 37 °C. The number of colonies was quantified as colony forming units (CFU) per mL of bacterial culture. The tests were performed in triplicate.

### 2.6. Data Analysis

Data were analyzed as viable bacterial cell curves over time. CFU were represented as Log 10 per milliliter (CFU/mL) versus time in minutes. Bactericidal activity was defined as a reduction of ≥3 Log 10 (CFU/mL) in relation to the initial population, while bacteriostatic activity corresponds to a decrease of <3 Log 10 (CFU/mL) in relation to the initial population of each strain.

## 3. Results

### 3.1. Phytochemical Components of S. queretaroensis Peel Aqueous Extract

The phytochemical analysis of the *S. queretaroensis* peel aqueous extract qualitatively revealed the presence of saponins, glycosides, and flavonoids in appreciable amounts, while alkaloids, tannins, and phenols were present in moderate quantities (Table 1).

### 3.2. Synthesis and Characterization of S. queretaroensis-Mediated Silver Nanoparticles

**Figure 1:**
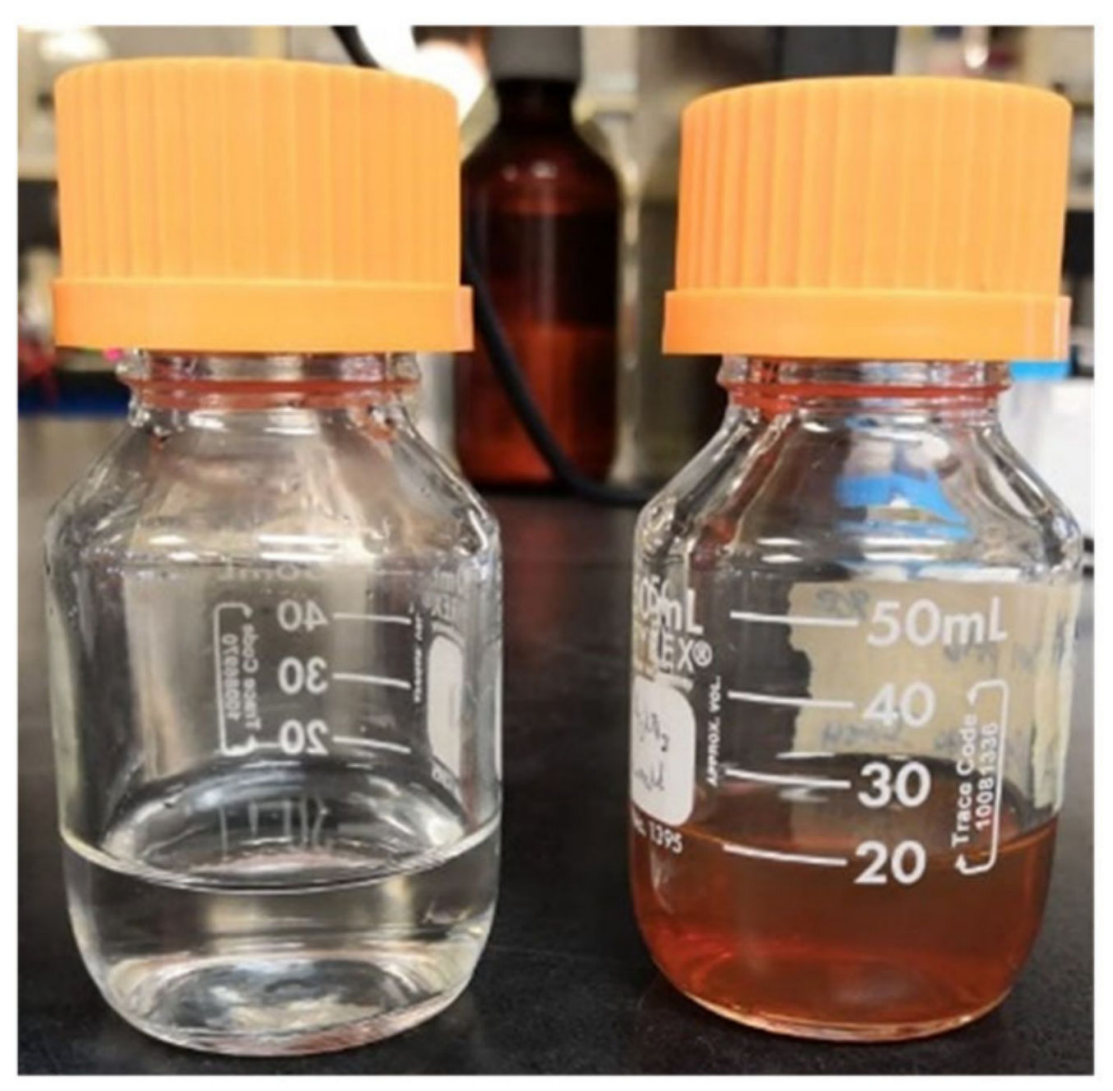
Biosynthesis of silver nanoparticles mediated by aqueous *S. queretaroensis* peel extract. Silver nitrate salt solution (colorless) converted into a light-yellow colored solution that later changed to a dark reddish-brown color.

**Figure 2:**
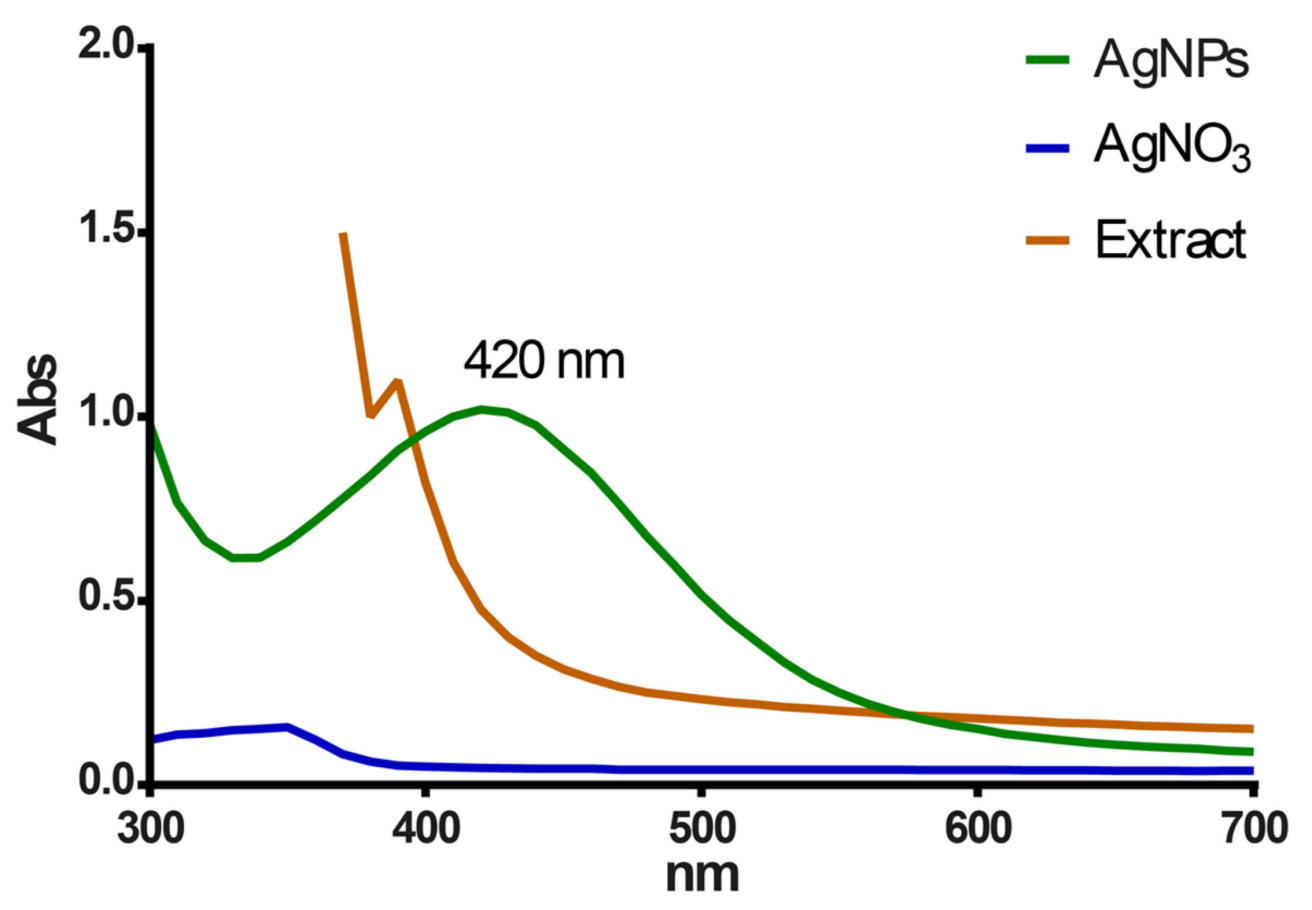
UV-vis absorption spectra of biosynthesized silver nanoparticles, aqueous *S. queretaroensis* peel extract, and silver nitrate salt solution.

The *S. queretaroensis* peel aqueous extract interacted with the silver nitrate salt solution (colorless) forming a light-yellow color solution that later changed to a dark reddish-brown color, indicating the reduction of ionic silver to reduced silver and the subsequent synthesis of AgNPs (Figure 1). A broad peak near 420 nm in the UV-visible spectra confirmed the synthesis of *S. queretaroensis*-mediated AgNPs (Figure 2), while the *S. queretaroensis* peel aqueous extract showed strong absorption below 400 nm. The colorless silver nitrate solution had no appreciable peak in visible light spectrum.

#### 3.2.1. FTIR Spectral Analysis

**Figure 3:**
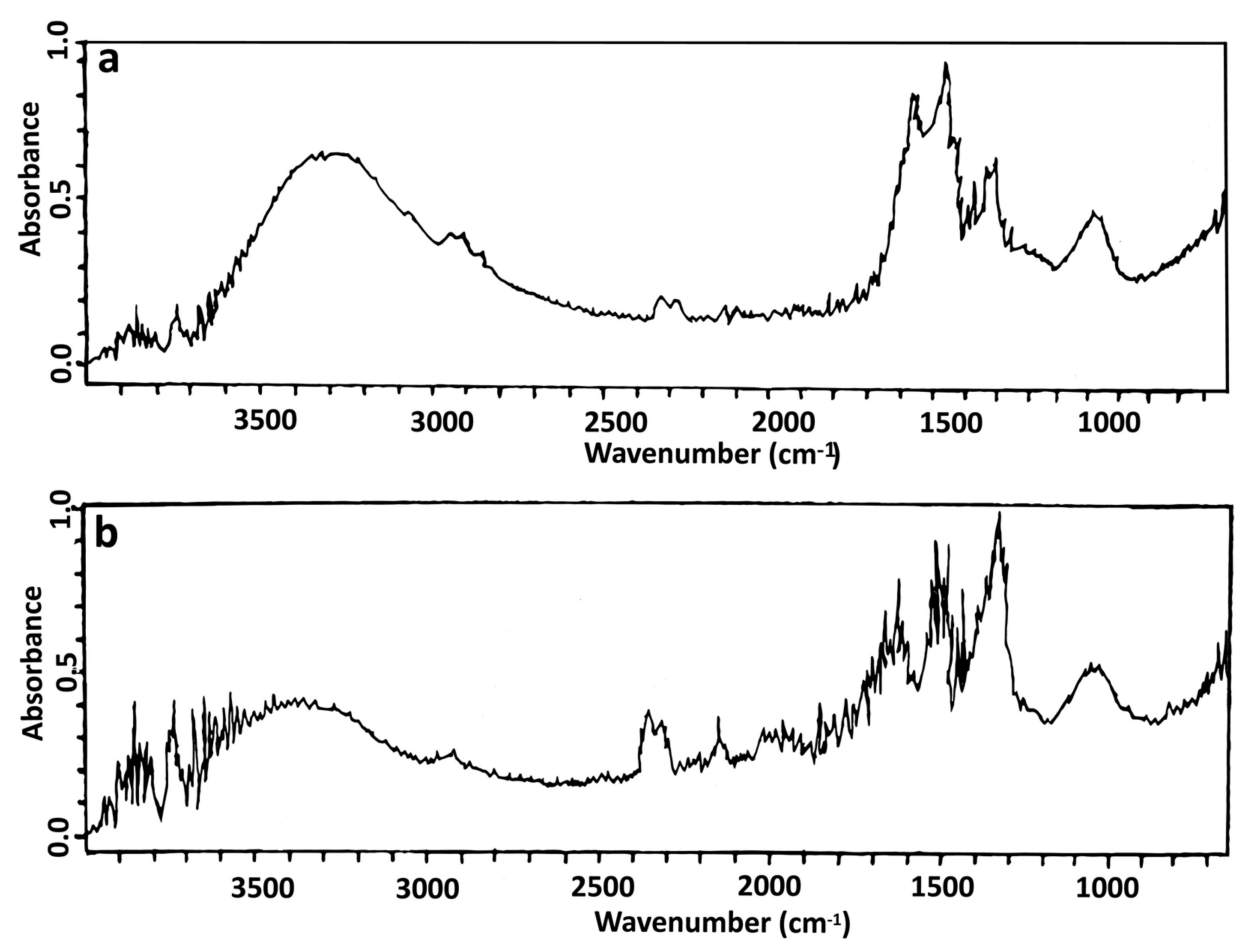
Fourier Transform Infrared Spectroscopy analysis of (**a**) *S. queretaroensis* peel aqueous extract and (**b**) biosynthesized silver nanoparticles.

FTIR analysis of nanoparticles compared to *S. queretaroensis* peel aqueous extract was performed to identify the biomolecules responsible for the reduction of silver nitrate of the biosynthesized AgNPs by *S. queretaroensis* peel aqueous extract. FTIR spectrum of the extract showed peaks at 3500–3200 cm^−1^ (Hydrogen-bonded O-H stretch characteristic of phenols), 1650–1585 cm^−1^ (C-C=C Symmetric stretch characteristic of flavonoids and aromatic rings), and a peak at 1100–1000 cm^−1^ characteristic of sugars such as fructose or glucose and C-O bonds within the alcohols (Figure 3a), while in the FTIR spectra of *S. queretaroensis*-mediated AgNPs, the band at 1650–1585 cm^−1^ was not present (Figure 3b).

#### 3.2.2. Particle Size Analysis

**Figure 4:**
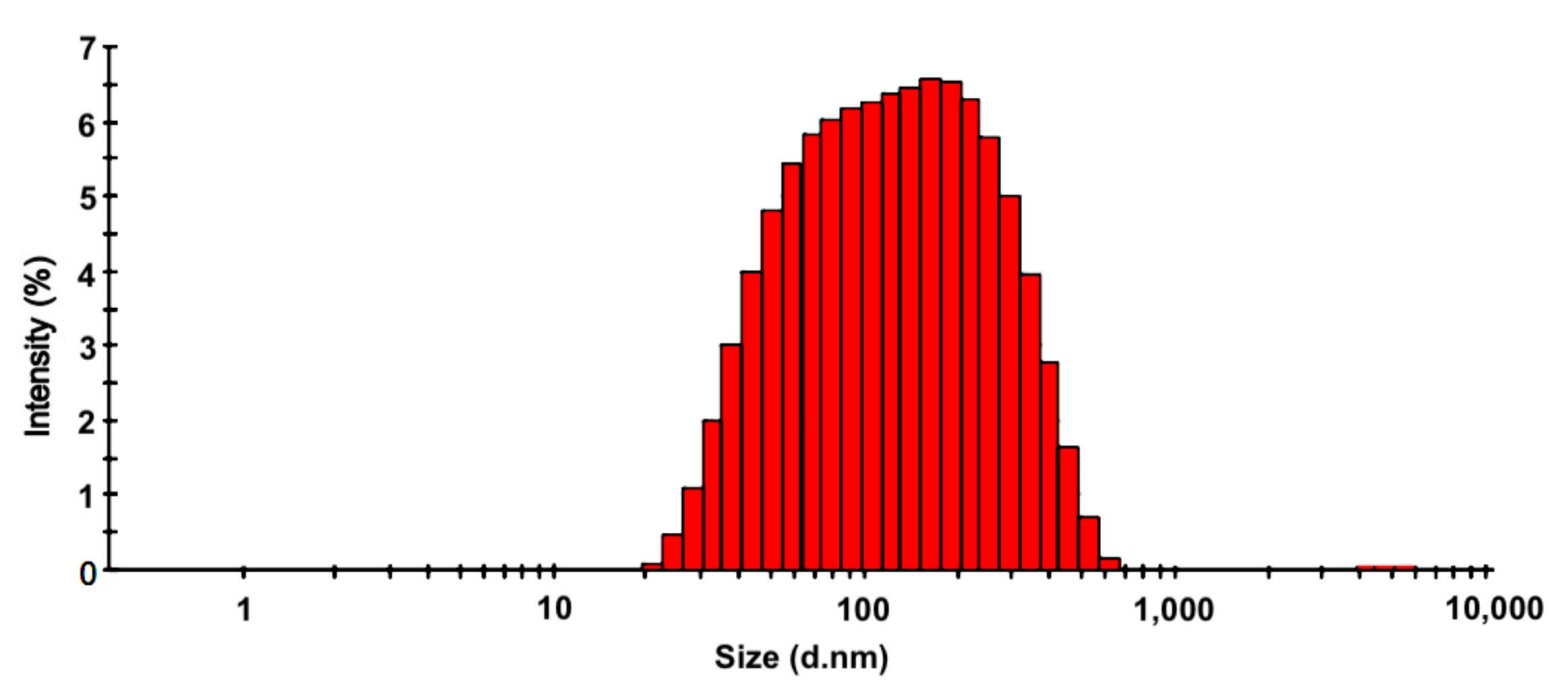
Size distribution of *S. queretaroensis*-mediated silver nanoparticles by dynamic light scattering analyses.

DLS analysis displayed a size distribution of the *S. queretaroensis*-biosynthesized silver nanoparticles that ranged between 20 and 600 nm (Figure 4) with a majority of AgNPs in the range of 60 to 200 nm and an average particle size of 98.96 nm.

#### 3.2.3. TEM Analysis

**Figure 5:**
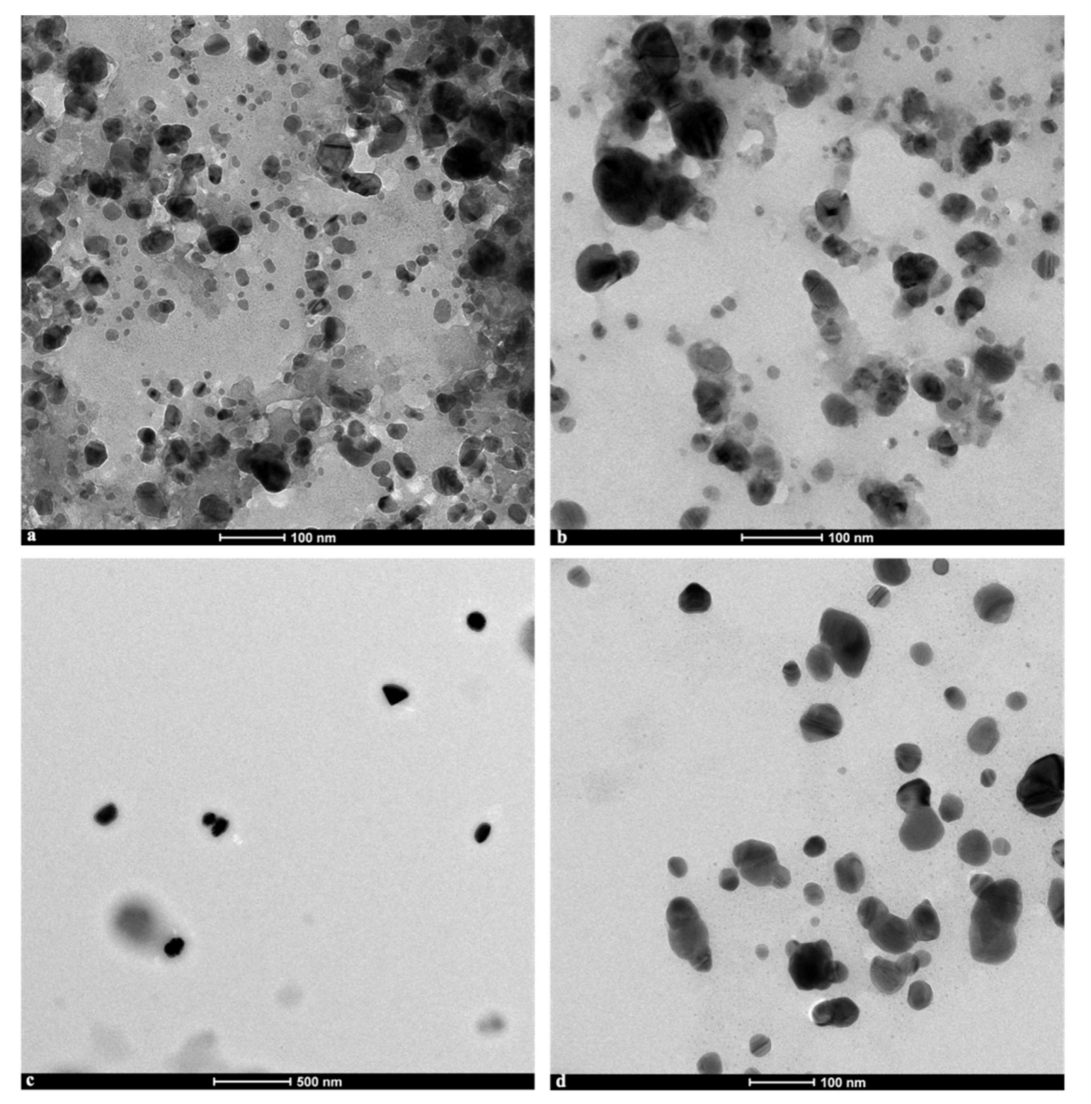
TEM micrographs of the silver nanoparticles biosynthesized by aqueous *S. queretaroensis* peel extract in colloidal solution at nominal magnifications of (**a**) 50,000×, (**b**) 62,000×, (**c**) isolated at a dilution of 1 in 50 at a nominal magnification of 11,000× and (**d**) in their pure form after being washed (at 50,000×).

Micrographs obtained by transmission electron microscopy to determine the shape and morphology of the *S. queretaroensis*-mediated AgNPs showed that the particles in colloidal solution (Figure 5a–c) and pure form (Figure 5d) were roughly spherical and uniform in shape and in addition did not create large agglomerates. 

### 3.3. Antimicrobial Activity of S. queretaroensis-Mediated AgNPs

#### 3.3.1. Minimum Inhibitory and Minimum Bactericidal Concentrations

The lowest concentration of the *S. queretaroensis*-mediated AgNPs to inhibit the growth of microorganisms ranged from 0.078 to 0.313 μg/mL. The gram-negative bacteria *S. enterica* and the yeast *C. albicans* showed a MIC value of 0.078 μg/mL while the gram-positive bacteria *S. aureus* and methicillin-resistant *S. aureus* (MRSA) along gram-negative *E. coli* showed MIC values of 0.313 μg/mL. Lastly, *P. aeruginosa* showed a MIC value of 0.156 ug/mL (Table 2).

The lowest concentration of biosynthesized AgNPs by *S. queretaroensis* peel aqueous extract required to kill microorganisms (no growth on the agar plate) ranged from 0.156 to 0.625 μg/mL. In the study, MBC for *S. enterica* and *C. albicans* were 0.156 μg/mL while *S. aureus*, MRSA and *E. coli* showed the MBC value of 0.625 μg/mL. The gram-negative *P. aeruginosa* showed the lowest MBC value of 0.313 ug/mL (Table 2).

The mean values of MIC and MBC for all microorganisms tested in this study was of 0.208 ± 0.118 μg/mL and 0.417 ± 0.235 μg/mL, respectively.

#### 3.3.2. Time-Kill Kinetics of AgNPs

**Figure 6:**
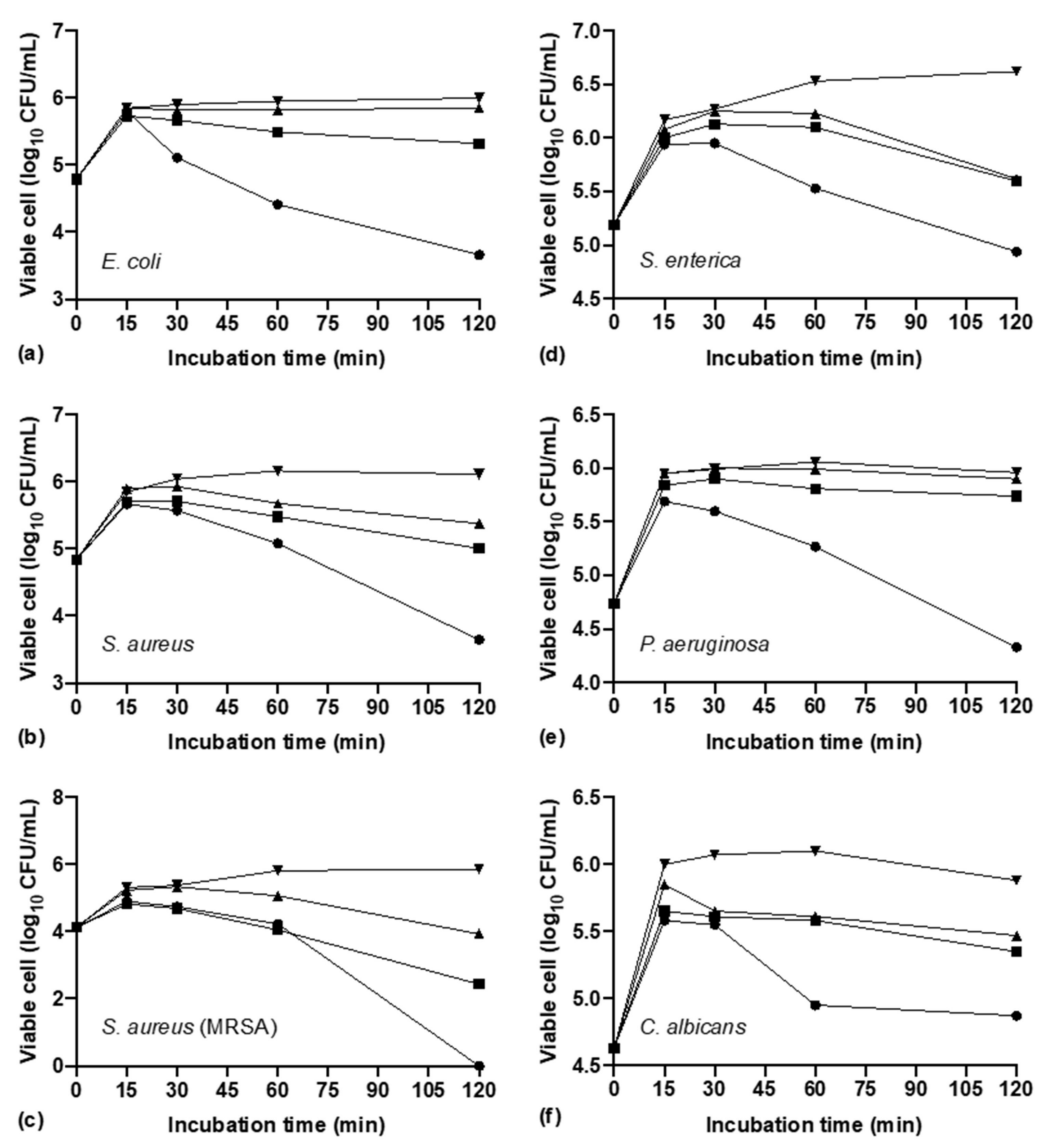
Time-kill kinetics of silver nanoparticles biosynthesized from *S. queretaroensis* against (**a**) *E. coli*, (**b**) *S. aureus*, (**c**) methicillin-resistant *S. aureus*, (**d**) *S. enterica*, (**e**) *P. aeruginosa* (**f**), *C. albicans* at three different concentrations (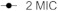, 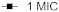, 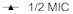, and 0
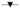

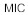
) added to media containing a starting culture.

AgNPs biosynthesized by *S. queretaroensis* tested at concentration of 2 × MIC exhibited a bactericidal effect after 2 h of incubation for all microorganisms tested in this study, reducing the log CFU/mL concentration regarding the log CFU/mL concentration of the first 15 min of incubation. *E. coli* and *S. aureus* showed a similar reduction of 2.34 and 2.46 logs (Figure 6a,b), while the strain methicillin-resistant *S. aureus* exhibited the highest reduction of 5.85 logs (Figure 6c). The lowest reductions of 1.68, 1.63 and 1.01 logs were for *S. enterica*, *P. aeruginosa*, and *C. albicans*, respectively (Figure 6d–f).

AgNPs evaluated at 1 × MIC still showed a bactericidal effect reducing 3.4 logs for methicillin-resistant *S. aureus*, 1.10 and 1.02 logs for *S. aureus* and *S. enterica* (Figure 6b–d) but for *E. coli*, *P. aeruginosa* and *C. albicans* the reduction was less: 0.681, 0.22 and 0.53 logs, respectively (Figure 6a,e,f).

When the AgNPs were tested at concentration of 1/2 × MIC for *E. coli*, *P. aeruginosa* and *C. albicans* exhibited a bacteriostatic effect as the log CFU/mL over time remained roughly the same as the log CFU/mL concentration after the first 15 min of incubation (Figure 6a,e,f).

## 4. Discussion

Pitaya fruit (*S. queretaroensis*) lack widespread commercial use because its nutritional and biological properties are not fully known; recently, the presence of betacyanins, betaxanthins, vitamin C, carbohydrates, and fiber of the different parts of the fruit have been reported. Now, in this study, the phytochemical analysis of the *S. queretaroensis* peel aqueous extract qualitatively showed the presence of saponins, glycosides, and flavonoids, in considerable quantities, while alkaloids, tannins and phenols were present in moderate amounts (Table 1). These new findings add value to the pitaya fruit since those compounds have biological activities that include anti-inflammatory [19], antioxidant [20], and wound healing [21] properties, and aid in cardiovascular diseases [22], acting as sedatives [23].

Medicinal plants are widely used as complementary and alternative medicine due to low cost and easy access. The presence of metabolites like flavonoids, glycosides, and alkaloids in the *S. queretaroensis* peel aqueous extract as noted from phytochemical color tests prompted us to test it as a reducer for silver nitrate.

Silver nanoparticles were successfully synthesized by mixing the *S. queretaroensis* peel aqueous extract with a silver nitrate salt solution, which was indicated by the color change of the reaction from a colorless to a reddish-brown color (Figure 1). Color change is due to the reduction of ionic silver from silver nitrate into silver nanoparticles mediated by biomolecules contained in the *S. queretaroensis* peel aqueous extract [24]. Besides the color change monitored visually, the UV-visible absorption spectrum of the *S. queretaroensis*-mediated AgNPs showed a broad peak at 420 nm, confirming the presence of AgNPs (Figure 2). The selected parameters for *S. queretaroensis*-mediated AgNPs biosynthesis was based on a review of different studies, from which a series of parameters were obtained that were supported by a response surface design and yielded a series of combinations that were tested (data not shown). Silver nitrate concentration and temperature were the most significant factors in the formation of nanoparticles.

Silver nanoparticles were characterized in this study visually (Figure 1), by UV-visible spectroscopy (Figure 2) and by TEM analysis (Figure 5) to determine its formation and by FTIR spectroscopy (Figure 3) to identify the metabolites responsible for the AgNPs formation, as well as the measurement of the nanoparticles size range by DLS analysis [25,26,27]. The FTIR spectrum obtained from the *S. queretaroensis* peel aqueous extract showed a characteristic peak of flavonoids at 1650–1585 cm^−1^ that was not present in the *S. queretaroensis*-mediated AgNPs (Figure 3), suggesting a possible biosynthesis mechanism: flavonoids hydroxyl groups bind to ionic silver (Ag^+^), acting as a reducing compound to form silver metal atoms (Ag^0^).

AgNPs synthesized by biological methods have shown a higher antibacterial effect regarding those synthesized by chemical methods [28]. The *S. queretaroensis* peel aqueous extract did not show any antibacterial activity at the same concentrations tested as *S. queretaroensis*-mediated AgNPs. Similar results have been reported where NPs have greater antimicrobial activity than plant extracts, and it has also been observed that some extracts show no activity as well [29]. An advantage that stands out from the use of nanoparticles in antimicrobial activity compared to plant extracts is that, by using a lower concentration of synthesized nanoparticles from plant extracts, it is possible to achieve an activity equivalent to plant extracts used at high concentrations [27].

MIC mean value for gram-negative bacteria was 0.182 ug/mL, while for gram-positive bacteria it was 0.313 ug/mL and 0.078 ug/mL for yeast. The nanoparticles biosynthesized with pitaya peel reported in this study showed a higher antimicrobial effect (Table 2) compared to AgNPs of size 20–60 nm obtained from the *Ferocactus echidne* cactus [7] tested at a concentration of 0.1 g/mL (>300,000 times higher than MIC values reported in our study) against *E. coli*, *S. aureus*, and *C. albicans*. Unfortunately, MIC values were not evaluated for a better comparison (1.8, 2.1 and 1.6 mm inhibition zones, respectively). The MIC and MBC values of 0.313 and 0.625 µg/mL of *S. queretaroensis*-mediated AgNPs on *E. coli* were 50–200 times lower compared to the MIC values reported by AgNPs biosynthesized from fruit pulp extracts of *Murraya koenigii* (MIC 16 µg/mL; MBC 32 µg/mL) and *Theobroma grandiflorum* (31 µg/mL; MBC 125 µg/mL) [30]. A similar result arose comparing the MIC and MBC values (0.313 and 0.625 µg/mL) of *S. queretaroensis-*mediated AgNPs for *S. aureus* regard to AgNPs biosynthesized by *Spondias mombin* (31 µg/mL: MBC: 62 µg/mL) and *Theobroma grandiflorum* (16 µg/mL: MBC 62 µg/mL) [30]. Even MIC an MBC values for the drug-resistant pathogen MRSA were 100 times lower than those AgNPs biosynthesized by *Murraya koenigii* (MIC 32 µg/mL: MBC 64 µg/mL) where nanoparticles had a smaller size (5–20 nm) compared to AgNPs biosynthesized by *S. queretaroensis* reported in this study [31]. Similarly, for *C. albicans*, the nanoparticles biosynthesized by *Euterpe oleraceae* showed MIC (8 µg/mL) and MBC (31 µg/mL) values 100–200 times higher than *S. queretaroensi*s-mediated AgNPs [24,30,31,32].

MIC values describe the antimicrobial effect at a concentration after a specific time, while the time time-kill kinetics assay evaluate the effect of different concentrations of MIC obtained values (i.e., 2 ×, 1 × MIC) throughout the time. The highest values of logarithm reduction showed by time-kill assay (Figure 6a–c) were obtained for the strain, methicillin-resistant *S. aureus* (2 × MIC and 1 × MIC reduced 5.85 and 3.41 logs) followed by *S. aureus* (2 × MIC reduced 2.46 logs) and *E. coli* (2 × MIC reduced 2.34 logs). The bacteriostatic effect by ½ × MIC coincides with a study report by Loo et al. [24], however, the microbicidal effect by AgNPs biosynthesized by *S. queretaroensis* at a concentration of 2 × MIC and 1 × MIC in this study is achieved in a shorted period of time (2 h).

It is well known that the antimicrobial activity of AgNPs is mainly attributed to the interaction between the negative charge on the cell membrane of microorganisms and functional groups on its surface and the positive charge of the nanoparticles. However, dynamic light scattering experiments, which measure Brownian motion and relates it to particle size, could suggest that the antimicrobial activity found in this study by AgNPs biosynthesized by *S. queretaroensis* peel is also related to the interruption of respiration and cell division cycles causing a lysis due to the AgNPs size since they have a larger area of contact with microorganisms [33].

Due to its antimicrobial effects at low concentrations, it is recommended to test more pathogens for microbicidal activity. Moreover, stability and toxicity tests should be conducted on these nanoparticles in order to obtain a safe and s product that can be used to clean living and inert surfaces and synthesize nanoparticles of *S. queretaroensis* peel from other metals besides silver.

## 5. Conclusions

Silver nanoparticles were successfully biosynthesized using the aqueous extract of the fruit peel of *S. queretaroensis* as a reducer. These nanoparticles tested at a low concentration displayed potent antimicrobial activity against gram-negative bacteria (*E. coli*, *S. enterica* and *P. aeruginosa*), gram-positive bacteria (*S. aureus* and *MRSA*) that cause foodborne illness, and the opportunistic medical pathogenic fungus *C. albicans*, which causes candidiasis. Therefore, these *S. queretaroensis*-mediated silver nanoparticles could be the basis for the formulation of biofilms for packaging products or as disinfectants for use on different surfaces.

## Figures and Tables

**Table 1:** Phytochemical components of *S. queretaroensis* peel aqueous extract.

Test Name	Results
Test for saponins	+++
Test for glycosides	++
Test for alkaloids	+
Test for tannins	+
Test for flavonoids	++
Test for phenols	+

+++: appreciable amounts ++: moderate amounts +: low amounts. The tests were performed in triplicate.

**Table 2:** Minimal inhibitory concentration and minimal bactericide concentration (μg/mL) of biosynthesized silver nanoparticles by *S. queretaroensis* peel aqueous extract.

Microorganism	MIC (μg/mL) *	MBC (μg/mL) *
Gram-Negative Bacteria		
*E. coli*	0.313	0.625
*S. enterica*	0.078	0.156
*P. aeruginosa*	0.156	0.313
Gram-Positive Bacteria		
*S. aureus*	0.313	0.625
*S. aureus* (MRSA)	0.313	0.625
Yeast		
*C. albicans*	0.078	0.156

* Tested in two-fold dilution steps (Starting at 10 µg/mL).

## Data Availability

The data underlying this article will be shared on reasonable request from the corresponding author.
